# Gene Expression Profiling in Tibial Muscular Dystrophy Reveals Unfolded Protein Response and Altered Autophagy

**DOI:** 10.1371/journal.pone.0090819

**Published:** 2014-03-11

**Authors:** Mark Screen, Olayinka Raheem, Jeanette Holmlund-Hampf, Per Harald Jonson, Sanna Huovinen, Peter Hackman, Bjarne Udd

**Affiliations:** 1 Folkhälsan Institute of Genetics and Department of Medical Genetics, Haartman Institute, University of Helsinki, Helsinki, Finland; 2 Neuromuscular Research Unit, Department of Neurology, University Hospital and University of Tampere, Tampere, Finland; 3 Department of Pathology, Fimlab Laboratories, Tampere University Hospital and University of Tampere, Tampere, Finland; 4 Department of Neurology, Vaasa Central Hospital, Vaasa, Finland; University of Minnesota Medical School, United States of America

## Abstract

Tibial muscular dystrophy (TMD) is a late onset, autosomal dominant distal myopathy that results from mutations in the two last domains of titin. The cascade of molecular events leading from the causative *Titin* mutations to the preterm death of muscle cells in TMD is largely unknown. In this study we examined the mRNA and protein changes associated with the myopathology of TMD. To identify these components we performed gene expression profiling using muscle biopsies from TMD patients and healthy controls. The profiling results were confirmed through quantitative real-time PCR and protein level analysis. One of the pathways identified was activation of endoplasmic reticulum (ER) stress response. ER stress activates the unfolded protein response (UPR) pathway. UPR activation was supported by elevation of the marker genes *HSPA5*, *ERN1* and the UPR specific *XBP1* splice form. However, UPR activation appears to be insufficient to correct the protein abnormalities causing its activation because degenerative TMD muscle fibres show an increase in ubiquitinated protein inclusions. Abnormalities of VCP-associated degradation pathways are also suggested by the presence of proteolytic VCP fragments in western blotting, and VCP's accumulation within rimmed vacuoles in TMD muscle fibres together with p62 and LC3B positive autophagosomes. Thus, pathways controlling turnover and degradation, including autophagy, are distorted and lead to degeneration and loss of muscle fibres.

## Introduction

Tibial muscular dystrophy (TMD, OMIM: #600334, Udd myopathy) is an autosomal dominant distal myopathy with a particularly high prevalence in the Finnish population [1]–[3]. The disease is caused by heterozygous mutations in the last two exons (Mex5-6) of the *Titin (TTN)* gene [4]. Five other phenotypes have been reported to be associated with C-terminal titin mutations: recessive limb-girdle muscular dystrophy type 2J (LGMD2J) [2], dominant hereditary myopathy with early respiratory failure [5], [6], recessive early-onset myopathy with fatal cardiomyopathy [7], core myopathy with heart disease [8] and centronuclear myopathy [9]. All Finnish TMD patients reported so far share the 11 bp deletion/insertion FINmaj founder mutation [1], [2], which results in C-terminal defects in the M-line region of the sarcomeric titin protein [2]. Several other mutations in the last two exons of *TTN* have been shown to cause TMD in patients of other European populations [4], [10], [11].

TMD is clinically characterised by atrophy and weakness in the muscles of the anterior compartment of the lower leg (*tibialis anterior*, *extensor hallucis longus* and *extensor digitorum longus*) [3], with typical onset between 35–55 years [12]. Homozygous FINmaj mutations cause the manifestation of the completely different, and much more severe, early onset LGMD2J (OMIM: #608807) [2], [3], [13]. TMD and LGMD2J patients do not have facial muscle weakness, dysphagia or clinically manifest cardiomyopathy. TMD muscle biopsies show myopathic–dystrophic morphology with rare fibre necrosis and frequent rimmed vacuolated fibres in affected muscles [3], [12], [14].

Titin molecules are integral to striated muscle structure and function by forming the backbone of the continuous third filament system of the myofibrils. The titin molecule stretches from the Z-disc to the M-line of the sarcomere. One of titin's core functions is to provide muscle elasticity by returning thin and thick filaments to their positions after contractions [15], [16]. The last Ig-domain (M10) containing the FINmaj mutation is adjacent to the differentially spliced [17] is7 domain that contains a calpain-3 (CAPN3) binding site [13]. Immunofluorescent (IF) microscopy of homozygous mutant muscles has revealed an absence of C-terminal titin epitopes from at least the three last domains M9, is7 and M10 [2]. Together with the loss of M-line CAPN3 and mislocalization of the C-terminal ligand obscurin, this suggests that the FINmaj mutation leads to a proteolytic cleavage of the titin C-terminus [13], [18], [19].

As the downstream molecular pathology caused by the FINmaj mutation is still largely unknown, the objective of this study was to identify relevant changes through global expression profiling of affected distal muscles from TMD patients. Significantly changed pathways were identified and confirmed through quantitative real-time PCR and protein level analysis of marker molecules.

## Materials and Methods

### Ethics statement

All patient biopsies were obtained with written informed consent and according to the Helsinki declaration. The study was approved by the institutional review board of Helsinki University Hospital.

### Muscle biopsies

All patients were diagnosed based on DNA mutation testing. Control and patient biopsies were obtained from the distal muscles of male and female patients in a range of ages (37–92; Table 1).

**Table 1:** Summary of patient and control muscle biopsies.

Expression array	qPCR	WB/IF/IHC	Biopsy	Diagnosis	Sex	Age at biopsy	Pathology
T1	rT1		EHL	TMD	M	53	Rimmed vacuoles
T2			GL	TMD	M	62	No rimmed vacuoles
T3			TA	TMD	M	48	Rimmed vacuoles
T4	rT2	TMD 5	EHL	TMD	M	65	Rimmed vacuoles
T5			SOL	TMD	M	52	Rimmed vacuoles
T6			TP	TMD	M	52	No major pathology
T7			TA	TMD	M	52	Rimmed vacuoles
	rT3		EHL	TMD	M	92	Not known
	rT4		TA	TMD	M	73	Rimmed vacuoles
	rT5		TA	TMD	M	78	Rimmed vacuoles
		TMD 1	EHL	TMD	M	62	Rimmed vacuoles
		TMD 2	TA	TMD	M	55	Rimmed vacuoles
		TMD 3	GM	TMD	M	67	Rimmed vacuoles
		TMD 4	EDL	TMD	F	50–55	Rimmed vacuoles
		TMD 6	TA	TMD	M	44	Rimmed vacuoles
C1	rC1		TP	Control	M	76	Normal
C2			EHL	Control	M	76	Normal
C3	rC2		TA	Control	M	80	Normal
C4	rC3		EHL	Control	M	80	Normal
C5			SOL	Control	M	76	Normal
		Ctrl 1	TA	Control	M	37	Normal
		Ctrl 2	TA	Control	F	48	Normal
		Ctrl 3	THP	Control	F	91	Normal

*Tibialis anterior* (TA), *tibialis posterior* (TP), *gastrocnemius lateralis* (GL), *gastrocnemius medialis* (GM), *soleus* (SOL), *extensor hallucis longus* (EHL), *extensor digitorum longus* (EDL), thigh posterior (THP), immunofluorescence microscopy (IF), western blotting (WB) and immunohistochemistry (IHC). The expression array controls (C1, C2 & C5) and (C3 & C4) were collected from amputation material from the distal lower limb muscles of two individuals.

### RNA preparation

RNA was extracted from frozen muscles samples using RNAeasy mini kit's (Qiagen, USA) recommended protocol. The RNA quality was assessed using a 2100 Bioanalyzer and the Eukaryote total RNA Nano series II program (Agilent technologies, USA).

### Microarray analysis

3 µg of total RNA was used to generate double stranded cDNA. The cRNA was generated using either one- or two-cycle eukaryote target labelling along with controls supplied with the kit (Affymetrix, USA). The samples were biotin labelled and the cRNA randomly fragmented before hybridisation to Affymetrix U133plus2 microarrays following the manufacturer's instructions. The microarray chips were washed and stained using Affymetrix Fluidics Station and scanned using a GeneChip Scanner 3000.

Expression values were determined using Affymetrix Microarray Suite 5.0 and GeneSpring GX version 11.0 (Agilent Technologies, USA) was used for pre-processing the data using the MAS5 algorithm. All probe sets with absent flags were removed from the data. The data was baseline transformed to the median of all samples. Differentially expressed genes were detected using an unpaired *t*-test with unequal variance (Welch correction). *P*-values were corrected using a Benjamini-Hochberg adjustment for multiple testing. Probe sets having a *P*<0.05 and at least a two-fold change were considered to be differentially expressed. The microarray data is available from the GEO website (http://www.ncbi.nlm.nih.gov/geo/) with accession number GSE42806.

### Pathway Analysis

Pathway analysis was done using Ingenuity Pathway Analysis software (www.ingenuity.com). The significance of each canonical pathway having more affected genes than expected by chance in the data set was determined by the Fisher's exact *t*-test (*P*<0.05).

### Quantitative real-time PCR

RNA was extracted from tissue biopsies using a Trizol (Invitrogen, USA) based protocol according to the manufacturer's suggestions. 1 µg of total RNA was reverse-transcribed to cDNA using SuperScript II Reverse Transcriptase (Invitrogen, USA) following the manufacturer's protocol. Quantification of the cDNA was performed using TaqMan based quantitative real-time PCR. The primers and probes used were the spliced form of *XBP1* (Hs00231936_m1), *HSPA5* (Hs00607129_gH), *ERN1* (Hs00980093_m1), *JUN* (Hs99999141_s1), *HSPB1* (Hs03044127_g1) and *GAPDH* (4333764F). 10 µl TaqMan master mix (Applied Biosystems, USA), 0.5 µl 1∶10 diluted cDNA and 2 µl of primer and probe set were used in a 20 µl total reaction volume. Amplification and detection were performed using the ABI 7500 system (Applied Biosystems, USA). The PCR thermal conditions were 50°C for 2 min, 95°C for 10 s and 60°C for 1 min. Each sample was performed in triplicate and the expression was normalised to *GAPDH* using standard curves for each gene on the same plate.

### Statistical analysis

Statistical significance of the quantitative real-time PCR and western blotting results were calculated using an unpaired *t*-test (*P*<0.05).

### Western blotting

Muscle biopsies were homogenized with 19 volumes of sample buffer containing 4 M urea and 4% SDS at 100°C for 5 min. Samples were resolved using 12% SDS-PAGE gels with 4% stacking gels and transferred to PVDF membranes (Bio-Rad Laboratories, USA). Membranes were blocked in TBST 5% milk for 1 h at +4°C. Primary and secondary HPR-conjugated antibodies were incubated at +4°C for 1 h each, followed by 6× TBST (5 min) washes. Antibodies were detected using the SuperSignal West Femto ECL substrate (Pierce, Thermo Scientific, USA) and captured using photographic film. Protein loading was assessed by staining of the post-blotting gel using Bio-Safe Coomassie Stain (Bio-Rad Laboratories, USA). Antibodies used are listed in Table S1. Quantitative analysis of western blotting bands was performed using ImageJ version 1.46f (http://rsb.info.nih.gov/ij/).

### Immunohistochemistry and immunofluorescence

Muscle biopsies were snap frozen in liquid nitrogen cooled isopentane to make 6 µm cryosections on SuperFrost (Kindler GmbH, Germany). The sections were fixed in 4% PFA for 10 min, permeabilized in 0.2% Triton X-100 for 10 min, and blocked in 5% BSA for 30 min at room temperature. The DAB immunohistochemistry (IHC) (Universal DAB detection, Ventana, USA) was performed using the BenchMark automated immunostainer following manufacturer's instructions. LAMP2 IHC signal was enhanced using an amplification kit (cat. 760-080, Ventana, USA). Herovici staining (a modified van Gieson stain) on frozen muscle sections was used for identification of the rimmed vacuoles. The immunofluorescence microscopy was performed as described earlier [20]. Antibodies are listed in Table S1.

## Results

### Expression profiling and pathway analysis

**Figure 1:**
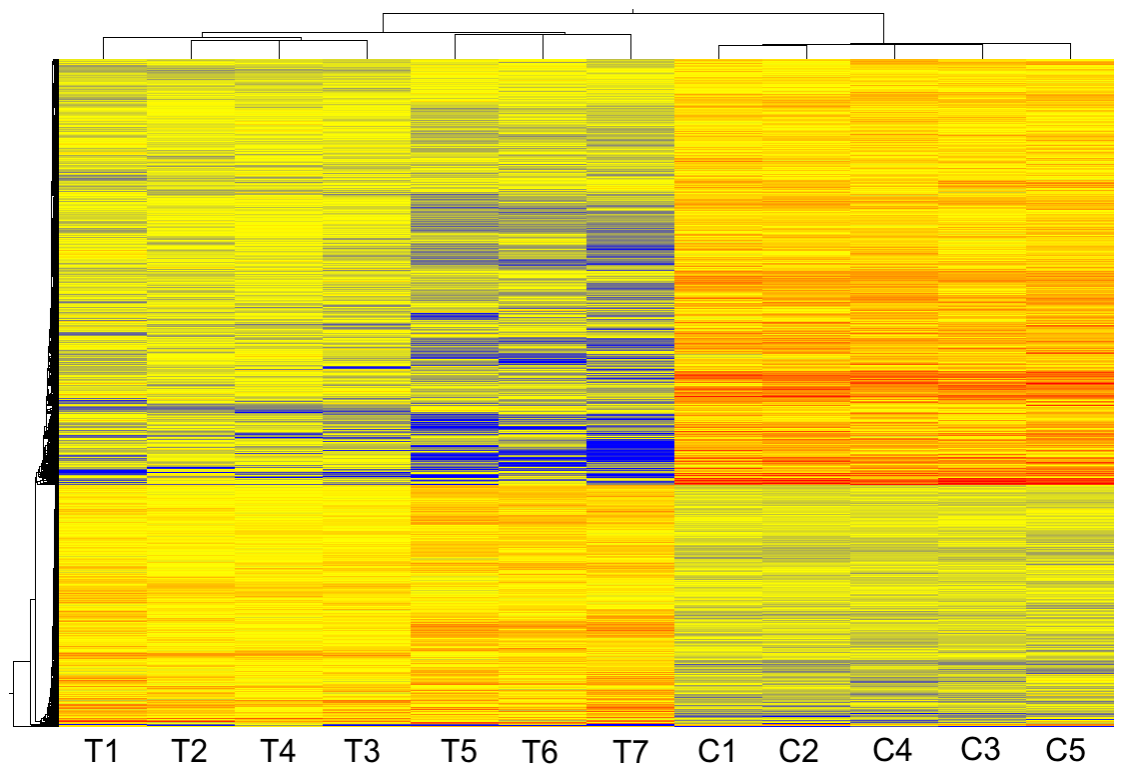
Hierarchal clustering of TMD and control expression profiles. Clustering of differentially expressed probes using centroid linkage and euclidean similarity in GeneSpring GX v11.0. The expression profiles of TMD (T1–T5) and controls (C1–C5) are represented as a heatmap (blue = low expression, red = high expression). A list of significantly changed probes is in Table S2.

**Figure 2:**
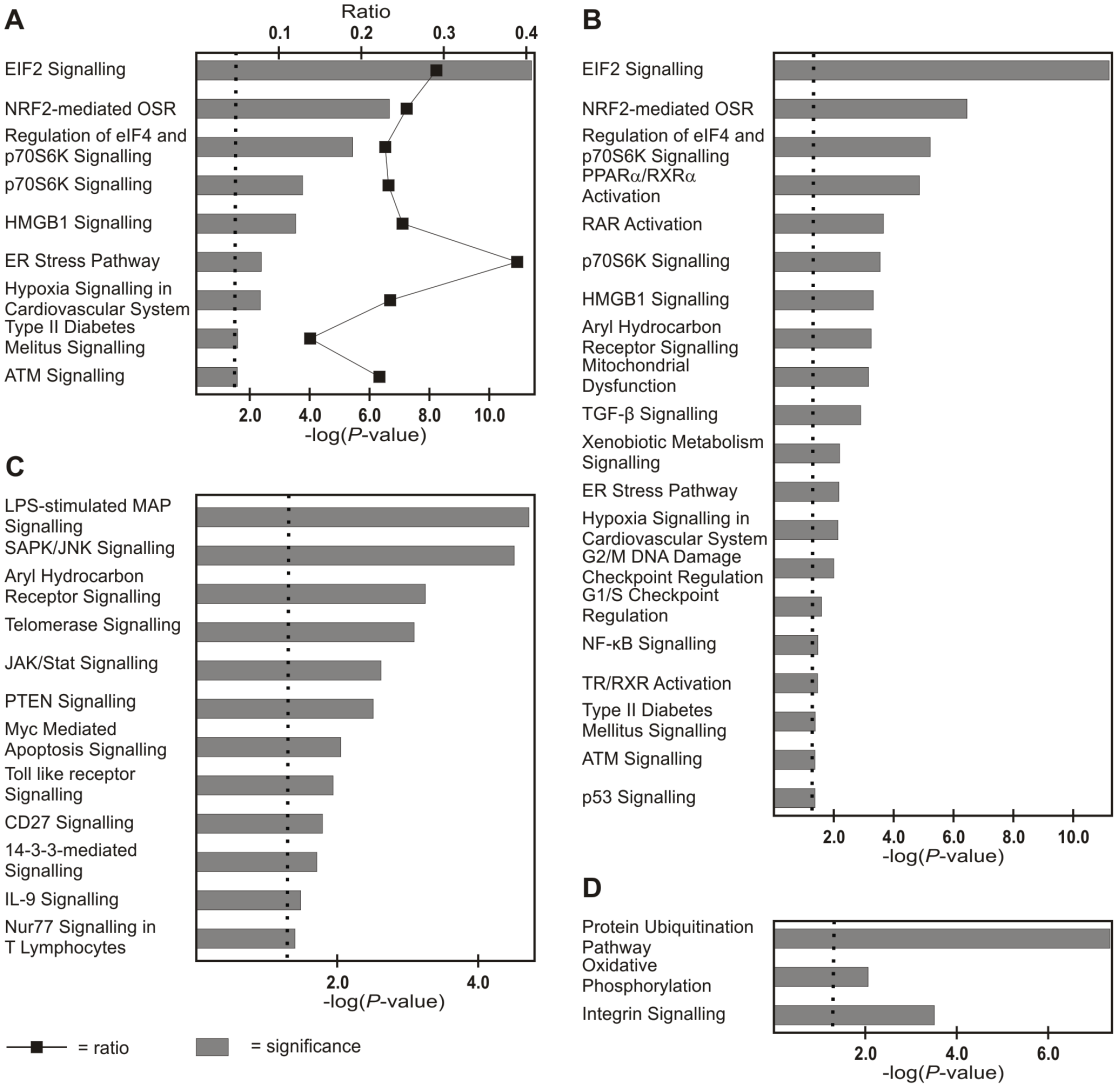
Ingenuity Pathway Analysis of TMD expression profiles. (A) Cell stress pathways affected in TMD also showing the ratio of significantly changed genes versus all genes in the given pathway (oxidative stress response; abbreviated to OSR). (B) Significantly altered cell stress and toxicity pathways, (C) apoptosis pathways, and (D) selected other pathways. The dotted line indicates the statistical significance threshold from a Fisher's exact *t*-test (*P*<0.05).

The expression profiles of seven TMD and five control biopsy samples were hierarchically clustered (Fig. 1). Significantly (*P*<0.05) changed probe sets in the TMD samples representing all differentially expressed transcripts are given in Table S2. Ingenuity Pathway Analysis (IPA) showed that endoplasmic reticulum (ER) stress response-, NRF2 mediated oxidative stress-, PTEN*-*, integrin- and EIF2- signalling pathways were among the most clearly affected pathways in TMD (Fig. 2A, 2B & 2C). There were also a range of pathway changes that have been identified in other myopathies with rimmed vacuoles such as; SAPK/JNK apoptosis- [21], p70S6K- [22], [23], protein ubiquitination- [23], [24] and mitochondrial dysfunction- [24], [25] signalling (Fig. 2B, 2C, & 2D).

### Verification of selected expression changes by quantitative real-time PCR

**Figure 3:**
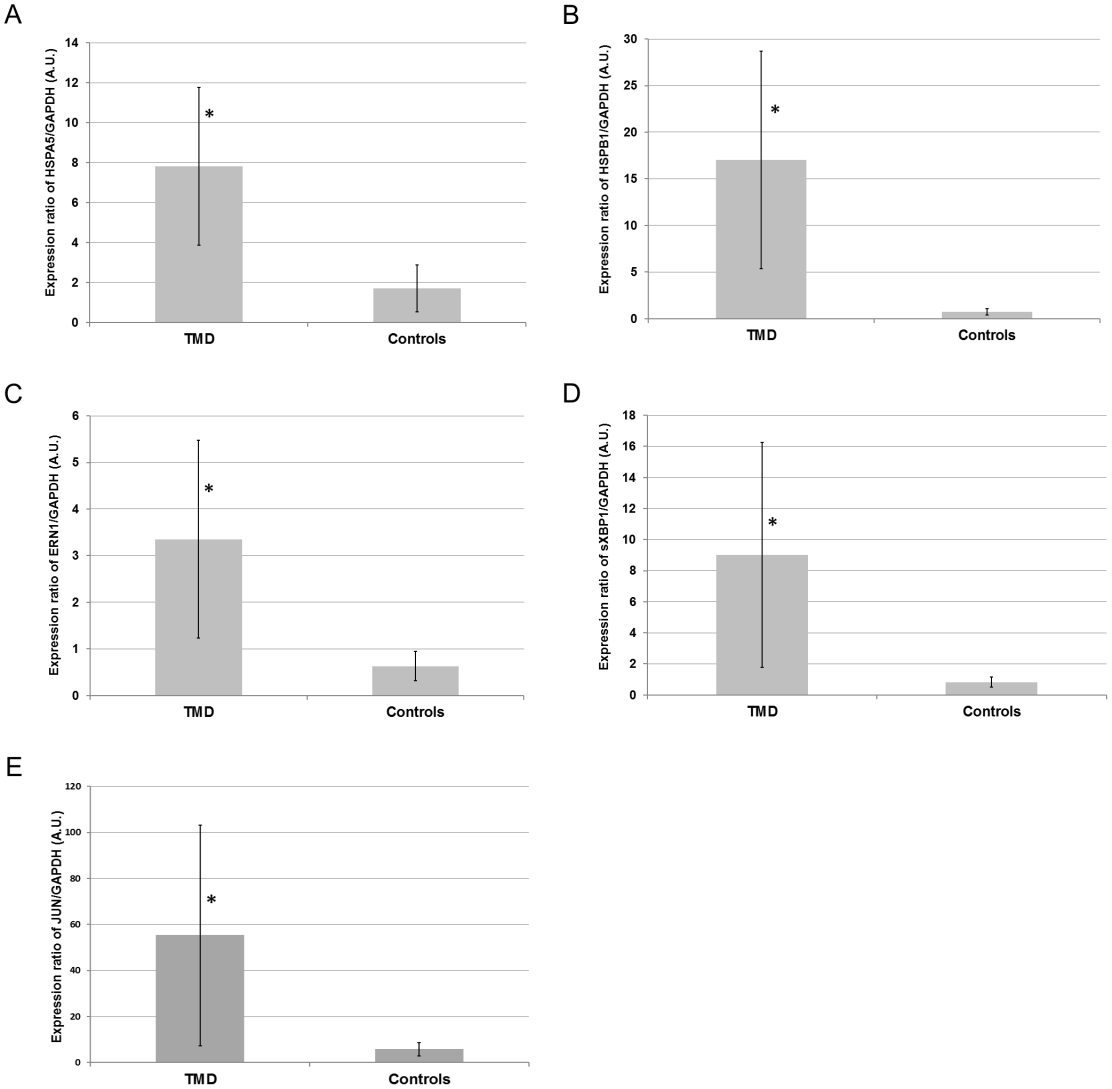
Quantitative real-time PCR analysis of key components of selected pathways. Expression of (A) *HSPA5*, (B) *HSPB1*, (C) *ERN1*, (D) spliced (s) *XBP1*, and (E) *JUN* normalised to *GAPDH* by quantitative real-time PCR from muscle biopsy total RNA (arbitrary units). *Indicates statistical significance in an unpaired *t*-test (*P*<0.05) comparison to the control group. The average and standard deviation of five TMD and three control biopsies each performed in triplicate are shown.

We chose to investigate ER stress response in TMD muscle since this pathway had the highest fraction of genes with significantly changed expression compared to controls. Unfolded protein response (UPR) is the main mechanism used to alleviate the effects of ER stress. HSPB1 proteins are involved in the ER stress response [26], whilst HSPA5 (BIP) [27], ERN1 (IRE1) [28] and the spliced form of XBP1 [29] are involved in the UPR pathway. Significant (*P*<0.05) up-regulation was observed in the expression of *HSPA5* (Fig. 3A), *HSPB1* (Fig. 3B), *ERN1* (Fig. 3C) and the previously described UPR specific *XBP1* splice isoform (Fig. 3D) in TMD samples versus controls.

The SAPK/JNK apoptotic signalling pathway was significantly (*P*<0.05) changed in the TMD samples compared to controls in IPA analysis. We analysed *JUN* (*CJUN*) expression by quantitative real-time PCR, as it is a final stage component of this pathway, and found significantly elevated expression levels of *JUN* in TMD samples (Fig. 3E).

### Analyses of UPR pathway components on the protein level

**Figure 4:**
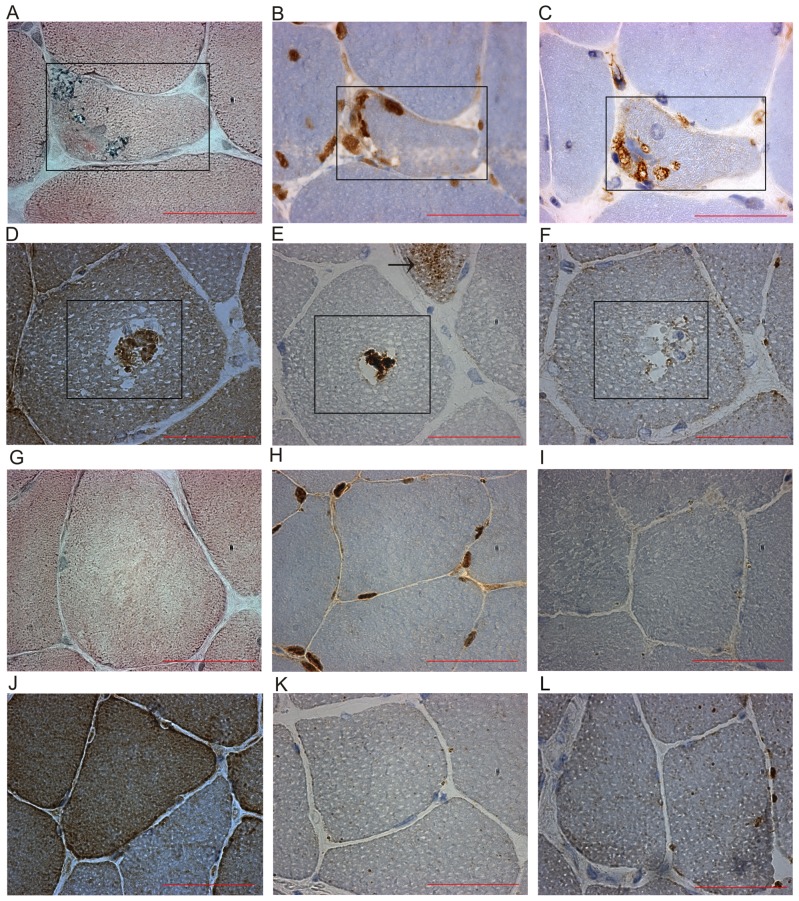
Histology and immunohistochemical analysis of TMD muscle biopsies. (A) Herovici histology staining of TMD muscle sections showing rimmed vacuolar regions within the fibres. (B) Immunohistochemical (IHC) analysis of TMD muscle sections stained for ubiquitin showing localised cytoplasmic ubiquitin containing inclusions in atrophic and rimmed vacuolated fibres and immunoreactivity at the edge of rimmed vacuoles. (C) Autophagosome marker LC3B staining showed strong immunoreactivity within the rimmed vacuolar regions. (D) VCP staining showed immunoreactivity in bodies within a large subset of rimmed vacuoles. (E) p62 staining showed immunoreactivity in rimmed vacuoles and granular immunoreactive dots (shown by an arrow) in an atrophic fibre. (F) Lysosomal protein LAMP2 staining showed no overall increase in rimmed vacuolated fibres. Two separate TMD fibres (A–C and D–F) with rimmed vacuoles in serial sections are shown. Controls of (G) Herovici staining, (H) ubiquitin, (I) LC3B, (J) VCP, (K) p62 and (L) LAMP2 IHC are included for comparison. Sections are from samples TMD 6 and Ctrl 3 in Table 1. Scale bar 50 µm.

**Figure 5:**
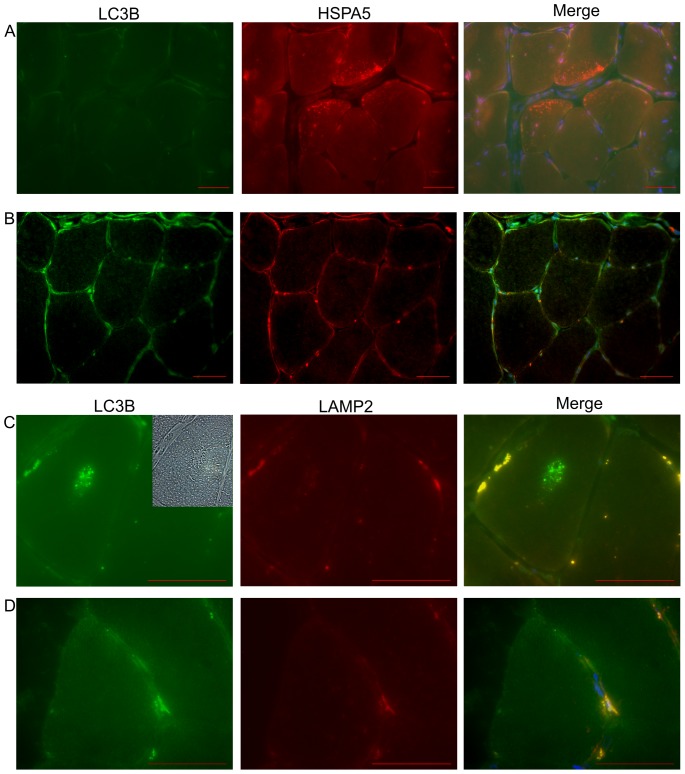
Immunofluorescent microscopy of TMD and control biopsies. (A) HSPA5 staining showed the presence of cytoplasmic granular dots in non-vacuolated TMD muscle fibres. The LC3B channel showed the area has no vacuolated fibres and no autofluorescent material present. (B) A control of HSPA5/LC3B double staining was included for comparison. (C) LAMP2 and LC3B double staining of a TMD muscle fibre showed a representative rimmed vacuole with massive accumulation of LC3B inside and negligible signal from LAMP2 around the edge of the fibre. The top right corner of the LC3B image showed a rimmed vacuole in bright field. (D) A control of LAMP2/LC3B double staining was included for comparison. All images are nuclear counterstained with DAPI (blue signal). Sections are from samples TMD 6 and Ctrl 3 in Table 1. Scale bar 50 µm.

**Figure 6:**
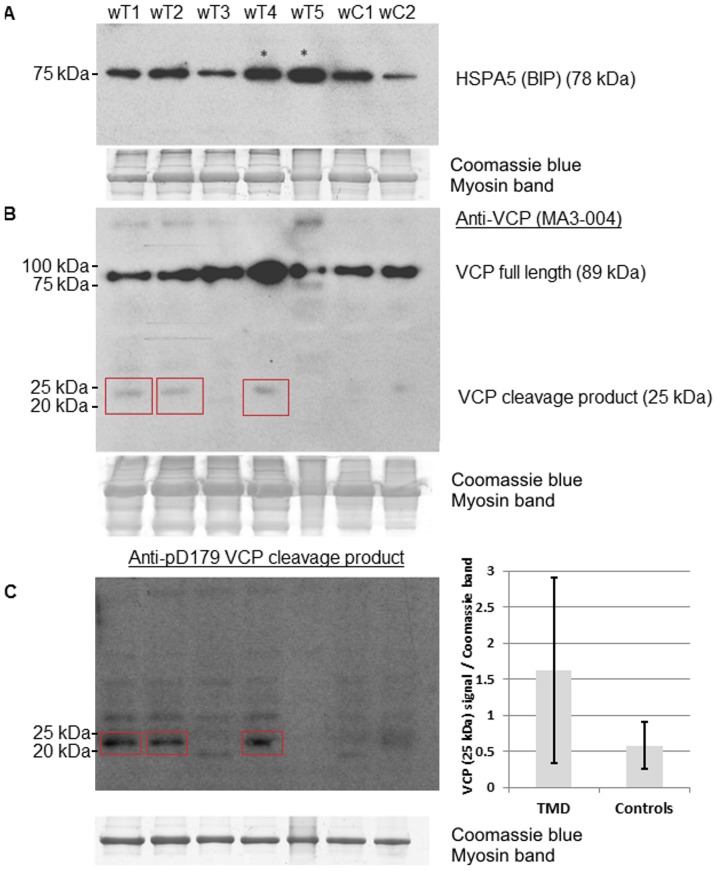
Protein level analysis of UPR and ERAD in TMD biopsies. (A) Western blotting showed HSPA5 increases in two in five samples when compared to controls. (B) Immunoblotting VCP showed an increase in full length VCP in two TMD samples and an extra band (∼70 kDa) just below the full length VCP in a third sample. Bands at approx. 25 kDa (red boxes) are observed in three of the TMD samples. (C) Samples were also blotted with p97D179 antiserum specific for VCP cleaved at D179 and show bands at approx. 25 kDa in three of the same samples. Total protein content is shown by the myosin band in Coomassie blue staining and showed equal sample loading. A graph of the quantification of the VCP cleavage product (25 kDa) signals normalised to the Coomassie myosin band showed a trend towards being increased in TMD in a *t-*test (*P* = 0.077).

Numerous rimmed vacuoles were identified in TMD biopsy sections by Herovici staining (Fig. 4A). We observed abnormalities in the ubiquitin-proteasome system (UPS) such as cytoplasmic ubiquitin containing inclusions (Fig. 4B) in the rimmed vacuolated fibres in IHC. We also observed the presence of HSPA5 granular cytoplasmic dots in non-vacuolated fibres by IF microscopy in TMD muscle biopsies (Fig. 5A), which were not observed in controls (Fig. 5B). This was in line with the up-regulation of *HSPA5* we found by quantitative real-time PCR analysis (Fig. 3A). By western blotting HSPA5 levels were increased in two out of five biopsies (Fig. 6A).

### Analysis of the ERAD pathway at the protein level

Abnormal or misfolded proteins within the ER are directed into the ER-associated protein degradation (ERAD) pathway. VCP is involved in proteasome-autophagy crosstalk [30], [31] and is a core component of the ERAD pathway [32]. In all 5 TMD biopsies tested by IHC, we observed a variable frequency of VCP-positive material connected with rimmed vacuoles (Fig. 4D). Abnormal VCP immunoreactivity was present as bodies in over half of the rimmed vacuoles. VCP labelling was also found in a subset of nuclei in control and TMD sections but did not differ between disease and controls samples. Western blotting (Fig. 6B) showed an increase in full length VCP in two TMD samples and an extra band (70 kDa) below the full length VCP in a third sample. Intriguingly, an extra 25-kDa band was observed in three out of five TMD biopsies and not in the controls. These bands were identified as D179-cleaved VCP by staining with a specific antiserum [33] (Fig. 6C). However, by quantification the increased levels of the 25-kDa VCP cleavage product in TMD did not reach statistical significance (*P* = 0.077).

### Analyses of autophagy pathway components on the protein level

If a protein abnormality cannot be correctly handled and processed through UPR activation using the UPS for degradation, the proteins may be redirected to the autophagic pathway and degraded in the lysosomal system. We observed strong immunoreactivity of the autophagosome marker LC3B in the rimmed vacuoles and in the cytoplasm of very atrophic fibres (Fig. 4C). Accumulation of p62 and the autophagosome marker LC3B have been used to indicate abnormalities in the autophagic degradation pathways [34]–[36]. We observed p62 immunoreactivity in rimmed vacuoles and granular immunoreactive dots in many atrophic fibres (Fig. 4E). There was no overall increase in immunoreactivity in the lysosomal marker LAMP2 in TMD fibres or within the rimmed vacuoles (Fig. 4F & 5C) when compared to controls (Fig. 4L & 5D).

## Discussion

The expression profiling results showed that the samples clustered according to the disease diagnosis as expected. IPA showed that there were differences in distinct molecular pathways between the TMD and the control samples. We were able to confirm selected changes in the studied pathways through quantitative real-time PCR and protein level analysis in the TMD samples.

Pathway analysis indicated a change in the unfolded protein response (UPR) pathway in the TMD samples. This was also suggested by the induction of ER stress response gene *HSPB1* and UPR genes *HSPA5*, *ERN1* and the splice form of *XBP1* in quantitative real-time PCR. The HSPA5 protein also showed restricted areas of increased expression in IF microscopy. Many of the proteins studied showed large variations between samples, and these differences may be due to the patient's biopsy site, disease severity or age at muscle biopsy. UPR activation has been reported in other rimmed vacuolar myopathies with abnormal UPS or lysosomal degradation pathways [37]–[40], and mildly in a mouse model of desminopathy [41]. This suggests UPR may act as a common protective mechanism during myopathic protein based stress. FINmaj mutated titin appears to cause the activation of UPR and lead to accumulation of ubiquitinated proteins. The increase of ubiquitinated protein components in TMD muscle is unlikely to be related to CAPN3 dysfunction. A secondary CAPN3 defect is present in homozygous LGMD2J, but in heterozygous TMD patients CAPN3 expression, although variable, is typically within the normal range [13].

Excess of misfolded proteins in the ER may also activate the ERAD system [42]. A key component of the ERAD pathway is VCP which, among others, retro-translocates unfolded proteins from the ER into the cytosol for degradation by the UPS [43]. Primary mutations in VCP cause a muscle disease with rimmed vacuolar pathology [44], [45]. VCP has also been reported to regulate ubiquitin-containing autophagosome maturation during myopathic proteomic stress [30], [31], and may therefore be involved in autophagosomal activation in TMD muscle. VCP accumulates inside of rimmed vacuoles suggesting that it may have a role in TMD myopathology with similarities to other neurodegenerative proteinopathies such as VCP-mutated myopathy [44], [45]. Western blotting showed an increase in two out of five biopsies and an extra band beneath the full length form in a third biopsy. In addition, we observed unconventional anti-VCP positive bands of approx. 25 kDa in size in 3 of 5 TMD samples. These ∼25 kDa bands were subsequently confirmed to be VCP cleavage products by specific antiserum [33]. The expression of these VCP cleavage fragments has been reported to impair degradation by the UPS system in neuroblastoma cells [33], but further studies are required before this pathway can be linked to the pathomechanism of TMD.

The p62 protein acts as an auxiliary autophagy factor by directly binding ubiquitinated proteins and LC3, to facilitate their degradation by autophagy [35]. However, p62 also shuttles ubiquitinated proteins to the proteasome for degradation [46] and has been found in protein aggregates in other neuromuscular and neurodegenerative disorders [47]–[49]. The accumulation of LC3B positive autophagosome material and p62 within the rimmed vacuolated regions of the degenerative muscle fibres suggests massive activation of compensatory autophagy mechanisms in TMD. Cytoplasmic increase of LAMP2 has been reported in atrophic fibres in other rimmed vacuolar myopathies [50]. However, there was no consistent increase of mature lysosomes based on LAMP2 staining's in atrophic rimmed vacuolated TMD muscle fibres. Apparently the increased induction of autophagy is not paralleled by increased autophagic flux and further processing, which may cause the observed massive increase of LC3B compartments.

*JUN*, a final stage component of SAPK/JNK apoptosis signalling pathway was significantly increased in quantitative real-time PCR analysis. In distal myopathy with rimmed vacuoles (DMRV) JUN has been shown to be increased in vacuolated fibres [21]. Nevertheless, whether increased apoptosis leads to a loss of muscle fibres in TMD remains unsettled. A previous study [13] has indicated that apoptosis may not be consistently increased in TMD muscle, although apoptosis is increased in the homozygotic LGMD2J [13] muscle. However, the muscle pathology of LGMD2J is different to TMD and does not show rimmed vacuolar changes.

This study, based on expression profiling and molecular pathology, has identified changes in several subcellular molecular pathways that are apparently involved in the TMD pathomechanism. These include activation of UPR accompanied by increased amounts of ubiquitinated proteins and altered autophagic degradation leading to atrophic and rimmed vacuolated degenerated fibres. Apparently a variety of entries, i.e. different mutant proteins, can result in similar changes in catabolic pathways that control protein turnover. The changes identified in this study are downstream secondary effects of the primary FINmaj mutation in titin. The exact molecular mechanisms that trigger UPR, ERAD and autophagic dysregulation as well as the other abnormally regulated pathways identified in this study need further characterization in order to identify steps that could be therapeutically useful.

## Supporting Information

Table S1 A list of antibodies used in western blotting, immunohistochemistry and immunofluorescence.
(DOC)

Table S2 A list of significantly changed expression array probes in TMD versus controls.
(XLS)
