# Supplementary material for: Gene Expression Profiling in Tibial Muscular Dystrophy Reveals Unfolded Protein Response and Altered Autophagy
Source: PLoS One. 2014 Mar 11;9(3):e90819. doi: 10.1371/journal.pone.0090819 (PMC3949689; doi:10.1371/journal.pone.0090819)
Supplement: Table S1 — A list of antibodies used in western blotting, immunohistochemistry and immunofluorescence. (DOC) [file pone.0090819.s001.doc]

**Table S1.** A list of antibodies used in western blotting, immunohistochemistry and immunofluorescence.

| Species | Type | Protein | Company | Clone / catalogue no. |
| --- | --- | --- | --- | --- |
| Mouse | Monoclonal | VCP | Thermo Scientific | MA3-004 |
| Mouse | Monoclonal | LAMP2 | Southern biotech | H4B4 |
| Rabbit | Polyclonal | LC3B | Cell Signalling | #2775 |
| Mouse | Monoclonal | HSPA5 (BIP) | BD Biosciences | 610978 |
| Rabbit | Polyclonal | Ubiquitin | Dako | Z0458 |
| Mouse | Polyclonal | VCP cleavage VAPD-179 | Halawani *et al*., 2010 | Personal gift |
| Mouse | Monoclonal | P62 | Santa Cruz | 28359 |
